# Adolescents’ attitudes towards e-cigarette in Tunisia

**DOI:** 10.1192/j.eurpsy.2021.2174

**Published:** 2021-08-13

**Authors:** R. Maalej, Y. Zgueb, A. Aissa, U. Ouali, F. Nacef

**Affiliations:** 1 Psychiatry A, Razi Hospital, Manouba, Tunisia; 2 Psychiatry A Department, Razi Hospital, Manouba, Tunisia

**Keywords:** adolescent, perception, Electronic Nicotine Delivery Systems

## Abstract

**Introduction:**

E-cigarette use has increased over the last years. Many studies have examined teenagers’ attitudes towards smoking, but did not examine adolescents’ attitudes towards e-cigarettes.

**Objectives:**

The aim of this study was to examine high school students’ attitudes regarding e-cigarette safety, addictive properties and social norms and to compare e-cigarettes perceptions among e-cigarette users and non-users in Tunisia.

**Methods:**

A survey was conducted with a sample of 234 students in Mohamed Ali high school in Sfax, a town in South of Tunisia, in February 2020. Socio-demographic data and questions about vaping and attitudes towards e-cigarettes were used to evaluate students’ perceptions towards e-cigarettes.

**Results:**

Among high school students aged 15 to 20, 58,8% have ever used e-cigarette, 38,3% had done so within the previous 30 days and 20,5 % were regular users of vapes. 53.8% of students believed that e-cigarettes are harmful. 78.4% of them thought they were less harmful than regular cigarettes and 50.5% thought they could be addictive, 45.4% of students believed e-cigarette smoking decreased anxiety and a third thought it made them sociable (33.3%) and confident (30.6%).Adolescents who used e-cigarettes had significantly more favorable e-cigarette attitudes than non-users: they believed they were less harmful than tobacco (p=0,019), they were not addictive (p=0,005), they decreased anxiety (p=0,001) and they made the user sociable (p<0,001) and confident (p=0,01).

**Conclusions:**

Our results suggest the need to provide teenagers with the correct information about e-cigarettes risks, and the balance risk-benefit of their use.

**Disclosure:**

No significant relationships.

